# Biological Actions of Artemisinin: Insights from Medicinal Chemistry Studies

**DOI:** 10.3390/molecules15031378

**Published:** 2010-03-08

**Authors:** Jian Li, Bing Zhou

**Affiliations:** State Key Laboratory of Biomembrane and Membrane Biotechnology, School of Life Sciences, Tsinghua University, Beijing 100084, China; E-Mail: l-jian08@mails.tsinghua.edu.cn (J.L.)

**Keywords:** artemisinin, *PfATP6*, heme, iron, mitochondria

## Abstract

Artemisinins have become essential antimalarial drugs for increasingly widespread drug-resistant malaria strains. Although tremendous efforts have been devoted to decipher how this class of molecules works, their exact antimalarial mechanism is still an enigma. Several hypotheses have been proposed to explain their actions, including alkylation of heme by carbon-centered free radicals, interference with proteins such as the sarcoplasmic/endoplasmic calcium ATPase (SERCA), as well as damaging of normal mitochondrial functions. Besides artemisinins, other endoperoxides with various backbones have also been synthesized, some of which showed comparable or even higher antimalarial effects. It is noteworthy that among these artemisinin derivatives, some enantiomers displayed similar *in vitro* malaria killing efficacy. In this article, the proposed mechanisms of action of artemisinins are reviewed in light of medicinal chemistry findings characterized by efficacy-structure studies, with the hope of gaining more insight into how these potent drugs work.

## 1. Introduction

Though accurate numbers are not known, it is estimated that approximately one million people die of malaria each year [1]. The emergence of malaria strains that exhibit resistance to most drugs in clinical use further intensifies this health problem. Forty years ago, efforts by a consortium of Chinese scientists, supported by Chinese government, to combat this disastrous disease led to the discovery of a powerful antimalarial drug, artemisinin. In contrast to Cinchona alkaloids such as quinine, which only kill mature parasites, artemisinin is effective in killing nearly all asexual as well as sexual stages of the parasites [2]. Most importantly, after nearly forty years of artemisinin use no appearance of clinically meaningful drug-resistant malaria strains has been observed. Recently, the World Health Organization (WHO) recommended Artemisinin Combination Therapies (ACTs) as first line treatment of uncomplicated *Plasmodium falciparum*. In addition to malaria, activity of artemisinin against other parasites such as Leishmania [3], Schistosoma [4,5] and Toxoplasma [6,7] have also been reported. Further activities of artemisinin include anti-viral [8] and anti-cancer properties [9,10], implicating its possible application in virus and cancer chemotherapy. One thing worthy of note is that the activities of artemisinin are two to three orders of magnitude less potent against most other pathogenic organisms and cancer cells.

Despite quick onset of action and high efficacy on malaria parasites, artemisinin has low solubility in both water and oil, making it difficult to administrate through intravenous injection. Another disadvantage of artemisinin is its short half-life after administration, as it can be metabolized mainly by CYP2B6 [11] into inactive metabolites. A further drawback is the relatively high recrudescence rate of infection with artemisinin monotherapy [12]. Development of new antimalarial drugs based on the artemisinin prototype for increased stability, higher efficacy and lower toxicity has been an area of active investigation [13]. Recently it has been reported that a single-digit dose of a new trioxane combined with mefloquine dydrochloride showed better antimalarial efficacy than the typical ACTs, which require repeated dose regimen of artemisinins and other antimalarial drugs [14]. Besides finding new and improved antimalarial candidates, these structure-efficacy studies have also aided in illuminating the mode of action of artemisinins.

## 2. The History of Artemisinin

As worldwide resistance of malaria parasites to existing antimalarial drugs such as chloroquine, quinine and mefloquine quickly emerged in the 1960s, it became urgent to search for more effective antimalarial drugs. Due to the Vietnam War and domestic requirements, a large-scale endeavor was initiated In China around that time to identify and isolate new antimalarial compounds. The most prominent outcome of these efforts is the discovery of artemisinin, a discovery made possible by a consortium of teams and labs from different institutes around China. Through screening many traditional Chinese medicinal plants and descriptions, it was shown that the wormwood plant, *Artemisia annua*, contains a highly effective antimalarial compound. Although mentioned a few times in several famous Traditional Chinese Medicine books, the first description of the antipyretic activity of water-brewed extraction of *Artemisia annua* was in a less well-known book, *A Handbook of Prescriptions for Emergency Treatments*, written by Hong Ge, an alchemist in the Jin dynasty (265-420 A.D.). Initial efforts in using extract of wormwood did not produce significant antimalarial results, presumably due to incorrect extraction methods. Many Chinese scientists contributed to the final isolation and characterization of artemisinin. This collaborative team effort eventually led to the discovery of a superior antimalarial drug: artemisinin, an endoperoxide situated within the backbone of a sesquiterpene lactone.

After the successful extraction of artemisinin from *Artemisia annua*, followed by its structural identification [15], artemisinin was introduced to the rest of the world in 1979 [12]. Because of its excellent antimalarial efficacy, unusual chemical structure and low yield from natural production, interest expanded in exploring methods of chemical synthesis, which resulted in the successful semi-synthesis of this compound from artemisinic acid [16] and a total synthesis method in 1983 [17]. Later, many other methods were developed, with different raw materials and routes. Biosynthesis has also been investigated for the heterologous expression of artemisinin. Amorpha-4,11-diene, a precursor to artemisinin, was produced with high levels in *E. coli* [18]. For a review of heterologous biosynthesis of artemisinin, see Arsenault [19].

**Figure 1:**
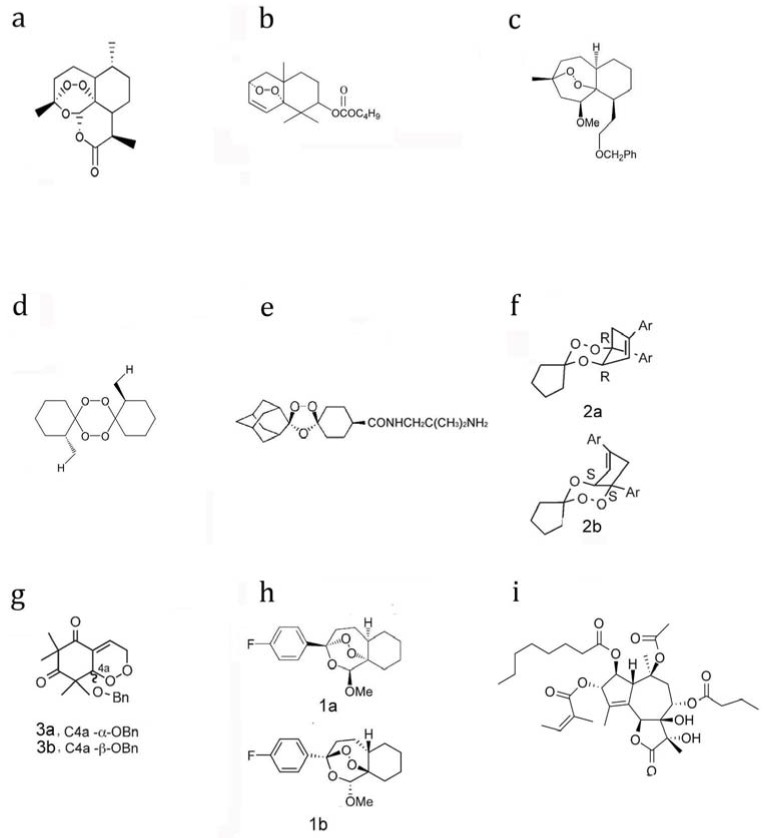
Structures of artemisinin and its analogues. (a) Artemisinin. (b) An early derived peroxide with less antimalarial potency. (c) An analogue with close structure to artemisinin. (d) An antimalarial tetraoxane. (e) OZ277. (f, g and h) Enantiomers with similar activities against malaria parasites. (**i**) thapsigargin.

After the discovery of the artemisinin prototype (Figure 1a), medicinal chemists have devoted much effort to trying to improve the efficacy of artemisinin. These early works produced several fruitful compounds now in clinical use. They are collectively known as artemisinins modified at the C_10_ position and are now standard drugs for treating malaria. Intriguingly, there is a good correlation between efficacy and the hydrophobicity/hydrophilicity ratio, with a peak ratio of logP centering around 2.7 [20]. In addition, multiple methods have been developed to make the administration of these derivatives more convenient [21]. Further medicinal chemistry studies based on the essential endoperoxide of artemisinin have led to the development of structurally simpler peroxide, trioxane, tetraoxane and certain other derivatives, which have opened a new page in antiplasmodial history. 

## 3. Physical and Chemical Properties of Artemisinin

Artemisinin has poor solubility in either water or oil, and instead, it is soluble in many aprotic solvents. In contrast to the general concept that molecules containing endoperoxides are susceptible to decomposition, artemisinin is amazingly thermostable: even when the temperature reaches its melting point at about 156-157 °C, no obvious decomposition is observed. However, further temperature increase to 190 °C leads to the breakdown of this molecule [22]. 

Artemisinin is unstable in the presence of alkali [23] or acid [24], resulting in the generation of mixed products. The endoperoxide moiety can be reduced by hydrogenation with Pd/C as the catalyst, giving rise to deoxyartemisinin [25], which has nearly no inhibitory effect on malaria. Zn/AcOH can also be used to hydrogenate the peroxy group and at a higher transformation rate [26]. In another hydroreduction reaction, however, the lactone group of artemisinin is transformed to a lactol in the presence of sodium borohydride, leaving the peroxy group unaffected [25]. It is this reaction that contributed to the development of early modified first generation artemisinin derivatives.

## 4. Artemisinins and Iron

Reaction of artemisinins with iron (nonheme or heme iron) received much attention for at least the following two reasons: artemisinin contains a peroxy group, which is reminiscent of the Fenton reaction, wherein H_2_O_2_ is catalyzed by ferrous iron or cupric copper to produce the HO^.^ free radical. It was proposed that free radicals generated from iron and artemisinin reaction mediate the antimalarial action of artemisinin [27,28]. In addition, a high concentration of heme was formed during hemoglobin digestion by the parasites [29], which may facilitate this decomposition reaction. Large amounts of research, including *in vitro* chemical investigations and *in situ* studies, were dedicated to the analysis of interaction between iron and artemisinins. 

Iron-mediated cleavage of artemisinin endoperoxide bridge was first proposed by the Meshnick group, who isolated heme-artemisinin adducts from artemisinin-treated *P. falciparum* [30] and by using a cyclic voltammetry method [31]. The authors then suggested that iron might play some role in the action of artemisinin, which could be achieved by activating artemisinin to free radicals. Interaction between iron and artemisinin was further revealed by EPR using the spin trap DMPO (5,5-dimethyl-1-pyrroline-*N*-oxide): artemisinin generated free radicals in an iron-dependent manner [32]. Artemisinin decomposition by iron initially produces oxy radicals with subsequent electronic rearrangements into carbon-centered radicals [33]. Based on these early and other results, models have been built to elucidate the free radical generation pathways of artemisinin after iron-mediated decomposition [34]. Nevertheless, the source of catalytic iron in malaria parasites is still unknown. Both non heme [32,35] and heme iron [36] were suggested to be able to cleave artemisinin, with respective experimental evidence to support those ideas. Many other studies related to the interaction of iron and artemisinin were also reported [37,38,39,40]. 

Some biological evidence does support iron involvement in the antimalarial action of artemisinins. Pyridoxal benzoylhydrazone and 1,2-dimethyl-3-hydroxypyrid-4-one, two hydrophobic iron chelators, were observed to be antagonistic to artemisinin and its derivatives [32]. Desferrioxamine, another iron chelator, also attenuated the effect of artemisinins [41]. Desferrioxamine can sequestrate free ferric iron and possibly alter intracellular ferric and ferrous iron equilibrium [42]. 

However, two alkylamino derivatives of artemisinin developed by the Haynes group showed superior antimalarial activity, but were much more inert to iron *in vitro* than other artemisinin derivatives [43]. The conclusion drawn by the authors is that reactivity with iron does not correlate with antimalarial activity, *i.e.*, compounds with strong iron activity may have feeble antimalarial activity, whereas compounds with little iron activity can display potent antimalarial properties. Yet, this piece of evidence does not rule out the involvement of iron in the action of antimalarial endoperoxides. For instance, although artemisone, an antimalarial drug derived from artemisinin, was shown to be inert to biomimetic ferrous sulfate *in vitro* and its antimalarial effect can only be moderately antagonized by DFO, these compounds still react with iron *in vitro*, albeit much more inefficiently, and it is not known what form of iron is involved *in vivo*. What is more, a significant antagonism was later observed between artemisone and DFP, a more lipophilic iron chelator [42]. To be fair, other effects of DFO were also suggested [44,45], and thus antagonism by DFO or DFP may have other interpretations.

In addition to iron, reactions mediated by other molecules have also been reported in which hydroperoxide was formed as the source of radicals [46,47]. In this hypothesis, heterolytic cleavage of the endoperoxide bridge occurred prior to the steps of rearrangement, resulting in the production of the hydroxyl radical, which damages cellular macromolecules. Another report also contradicted the role of iron in artemisinin action and suggested that the C radicals formed from artemisinins mediated by ferrous iron were not responsible for their antimalarial activities [48]. In this regard, although different pathways have been proposed, the physiological relevance of these different free radicals formed needs further evaluation. 

In our opinion, if iron is truly needed for the action of artemisinin, its involvement *in vivo* may be direct or indirect. One “indirect” scenario is that iron does not participate in the activation of artemisinin, but may involve in the propagation of ROS production, a downstream event, after the initial action of artemisinin. In other words, artemisinin’s action may initiate ROS production, and the ROS disrupt iron homeostasis, which in turn aggravates ROS production. Taken together, although it is widely accepted that iron participates in the action of artemisinins, the *in vivo* evidence is not yet absolute and some *in vitro* evidence suggests otherwise. Further, even if iron is involved, it is not known in which form and what stage or mechanism it is related to the action of artemisinins.

## 5. The Structure-Efficacy Relationship of Artemisinins and Other Endoperoxidic Antimalarials

To improve the pharmacological activity of the original artemisinin and to probe the structure-efficacy relationship, a series of endoperoxides based or modified on the 1,2,4-trioxane ring structure of artemisinin were synthesized, some with dramatically distinct backbones. The first generation of artemisinin derivatives are all simple esters and ethers of dihydroartemisinin modified at the C_10_ position. These analogues showed significant improvement over artemisinin in terms of efficacy and increased solubility. In addition to these derivatives, modification efforts were also devoted to obtaining new antimalarials with C_10_ or C_14_ substitutions [49,50,51,52]. All of these semi-synthetic compounds are derived using artemisinin as the starting material, and thus share similar backbones with artemisinin. During this phase of study it was reinforced that the endoperoxide in the backbone of artemisinin is the essential pharmacophore, whereas the 1,2,4-trioxane ring alone is not sufficient for the antimalarial activity [53]. In fact, ring A and lactone ring D are not essential for the antimalarial properties of artemisinin [54]. 

With this notion in mind, many groups have designed and synthesize diverse structurally simpler analogues. Initial efforts produced a cyclic peroxide (Figure 1b) in 1987, and its antimalarial activity was demonstrated *in vitro*, albeit with a more reduced potency compared to artemisinin [55]. Since then, large amounts of fully synthetic and simplified peroxide analogues were developed (Figure 1c and Figure 1d) [54,56,57]. These include synthetic cis-fused bicyclic 1,2,4-trioxanes such as fenozan B07 [58,59], the dispiro tetraoxanes [60], tricyclic simplified 1,2,4-trioxanes [61] and simple endoperoxide analogues [62]. Among these analogues, OZ277 (Figure 1e) received much attention and was even selected for antimalarial drug development [63]. For OZ277, the critical peroxidic pharmacophore is present within a 1,2,4-trioxolane rather than a 1,2,4-trioxane heterocycle. OZ277 was proved *in vitro* to have more potent antimalarial properties and greater chemical stability than artemisinin [63]. Intriguingly, diverse as the structures of these molecules may be, many of them manifest strong antimalarial properties, comparable to or even higher than that of the artemisinin prototype. One especially notable discovery in this area was the findings by O’Neill [64] and some other groups [65,66]. In these researches, pairs of enantiomerical endoperoxides (*Figure* 1f, g & h), with structures closely related or distant to artemisinin were chemically synthesized. All of these enantiomers showed similar *in vitro* IC_50_ values within each pair against malaria parasites, *i.e.*, not only enantiomers of structurally close artemisinin analogues, but also distinct 1,2,4-trioxane analogues in the fenozan series and simpler semisynthetic endoperoxides in the G3 series have similar antimalarial properties [64]. 

Taken together, the endoperoxide antimalarials group is truly unusual in that they are highly specific towards malaria parasites but the structures themselves can vary greatly. As is known, macro biomolecules are all chiral, and specific interaction between chemicals and proteins normally have strict structural (including chirality) requirements. An example illustrating this concept could be revealed in painkiller studies. There is a stringent requirement for the overall structure of morphine to bind to its receptor (activating the μ-opioid receptors in the central nervous system). Morphine enantiomers, dextrophan and levorphan, are associated with dramatically different activities: while levorphan is a potent pain killer, dextrophan is not at all active [67].

## 6. Mechanistic Insights from Chemistry Studies and Proposed Biological Models

One problem regarding the properties of artemisinin is its ultra specific interaction with malarial parasites without causing significant side effects in human cells. The key role of the endoperoxide bond suggests free radical is involved in its action, which is also supported by some *in vivo* data. However, H_2_O_2_ or other free radical generating agents, generally act promiscuously with no specificity. How is the specificity of artemisinin achieved? In principle, the specificity of artemisinins could arise from specific activation or specific damage, *i.e.*, the action specificity of artemisinins could be due to its specific activation in malaria or due to its specific targeting from activated artemisinin. 

Given the chemical findings mentioned above, we will review research evidence related to the biological mode of action of artemisinin and discuss these hypotheses from the chemical perspectives. Three major models are explained here. They differ in some fundamental aspects, but all of them are able to integrate the “indispensable” iron role into their respective mode of action. Thus none contradict the iron involvement. 

### 6.1. PfATP6 as the target

While previous studies regarding the mechanism properties of artemisinin have been mostly controversial, it should be noted that it is indisputable that the endoperoxide bond embedded in the backbone of the sesquiterpene lactone in artemisinin is essential for antimalarial properties. To explain the ultraselective action of artemisinin, it is generally inferred that a particular, as yet unknown target is involved [68]. This is understandable as many other drugs with specificity target highly selective biological macromolecules. 

Artemisinin was proposed to have a structure similar to that of thapsigargin (Figure 1i), which specifically binds and inhibits the sarcoplasmic/endoplasmic calcium ATPase (SERCA), in the absence of the endoperoxide bond. In order to explore the interaction of artemisinin and SERCA of *P. falciparum*, and the role of this interaction in its malaria killing [41], the only SERCA-type Ca^2+^-ATPase of *P. falciparum* (*PfATP6*) was expressed in *Xenopus laevis* oocytes. The potent and specific inhibitory effects of artemisinin on *PfATP6* were observed (*Ki* =150 nM), which were comparable to that of thapsigargin. While both artemisinin and thapsigargin are potent inhibitors of *PfATP6*, in a somewhat puzzling development, thapsigargin antagonized the antimalarial activity of artemisinin. It was interpreted that these two compounds have the same binding target in the parasites. Normally drugs with similar mechanism properties and effects, unless they have similar mechanisms and opposite effects, would not display antagonistic properties. Consistently, a later study [69] showed no antagonistic antimalarial activity exists between artemisinin with thapsigargin. Good correlation was also found between inhibitory constants against *PfATP6* and the ability to kill the cultured parasites among artemisinin and some of its derivatives. When cultured parasites were preincubated with artemisinin, labeling of the parasites using a fluorescent thapsigargin derivative was abolished, further supporting that artemisinin has the same targeting site as thapsigargin [41]. Based on this data, artemisinin was suggested to work through binding to *PfATP6* and disrupting its functions.

Later publications both for and against this theory were reported. Docking of artemisinins to *PfATP6* was performed showing that the binding abilities correlate well with malaria killing properties [70]. However, a recent, similar docking study of a series of antimalarial drugs, including artemisinin and its derivatives, quinoline based drugs and other peroxides into thapsigargin binding cleft, was performed, and no correlation was discovered between the binding affinity of these drugs to *PfATP6* and their antimalarial activities [71]. When Leu263 of *PfATP6* and the corresponding Glu255 of mammalian SERCA were altered to their respective counterparts, the inhibitory constants of artemisinin changed accordingly [72]. However, no artemisinin-resistant field isolates with these polymorphisms were identified. In an association study using field isolates from French Guiana, some isolates with S769N polymorphism in *PfATP6* showed obviously increased IC_50_ of artemether [73]. However, there is no data about the inhibitory efficacy of artemisinin on S769N *PfATP6*. In contrast, another field isolate containing *PfATP6* S769N was also reported and *in vitro* susceptibility assay revealed full sensitivity of this isolate to artemisinins [74], suggesting that polymorphisms, other than the *Pfatp6* one, exist that are responsible for the IC_50_ increase detected in some of the original isolates [73].

From a biochemistry point of view, it is not straightforward to reconcile the *PfATP6* model, or other models proposing how specific proteins are targeted and inactivated by artemisinins, with the chemical findings. In the *PfATP6* model, if artemisinin behaves similarly to thapsigargin, it would be expected that the binding of artemisinin to *PfATP6* would inhibit *PfATP6* activity. However, we know thapsigargin does not have an endoperoxide bond. To integrate the key role of the endoperoxide bond with the role of iron, it was proposed that artemisinin would undergo a specific non-covalent interaction with *PfATP6* after being activated by the catalytic quantity of labile iron, and irreversible damage by the formed free radicals would then be inflicted on this protein with the final death of malaria parasites. This hypothesis appears to be unsupported, although not excluded, by the discoveries made in medicinal chemistry studies wherein it has been found there is no antimalarial stereoselectivity of artemisinin derived enantiomers [64,65,66]. If a specific interaction between artemisinin and its target protein is involved, how can enantiomers of several artemisinin derivatives all have roughly identical potency within each pair? Besides, structure-efficacy results showed that many structurally distinct endoperoxides displayed potent antimalarial activities, but binding to *PfATP6* largely depends on the backbone structure rather than the peroxide bridge. More recently, the similarity of artemisinin to thapsigargin has also been questioned [75]. 

### 6.2. Heme as the activator and target 

The heme model proposes that artemisinin acts inside the vacuoles to inhibit malaria: after cleavage by heme, the resultant free radicals of artemisinin are supposed to randomly alkylate surrounding vacuolar targets such as heme. The heme model does not apparently contradict with what we know about the chiral properties of artemisinin because a strong and specific interaction between artemisinin and a protein target is not a prerequisite. To put it another way, chirality may not affect the interaction of heme and artemisinin.

Initially it was proposed that heme might be the target for activated artemisinin. According to this early version of the heme model, artemisinin works in a similar fashion to choloroquine: both work in vacuoles and inhibit malaria by interfering with heme detoxification. Supporting evidence for this model is listed below. When [^14^C]-artemisinin was added into erythrocytes infected with *P. falciparum*, radioactivity gauging results showed enrichment of artemisinin in hemozoin. By using high-performance liquid chromatography (HPLC), heme-artemisinin adducts were also isolated as in a test tube reaction [76], implying physiological relevance of the interaction between artemisinin and heme [36]. The capability of artemisinin to inhibit the proteolytic activity of digestive vacuoles was shown in both *in vitro* and *ex vivo* experiments in which artemisinin could potently inhibit heme polymerization [77]. Artemisinin was also demonstrated to be able to alkylate heme in infected mice [78]. In another report, heme in hemoglobin but not free heme was shown *in vitro* as the target of artemisinin; the antimalarial effect of artemisinin was proposed to be caused by impaired haemozoin formation from alkylated hemoglobin, as well as by creating a more reductive environment not suitable for haemozoin formation [79]. Chemically synthesized heme-artemisinin adducts inhibited the process of heme polymerization, resulting in the release of toxic heme/hemeart and the final death of malarial parasites [80,81]. Consistently, hemoglobin catabolism was found during the whole blood stage of malaria infection as well as ring stages [82,83,84]. Other associated studies also suggested a correlation between the heme binding ability and antimalarial activities of artemisinin derivatives [85,86,87]. 

However, some pieces of evidence argue against the heme target hypothesis. In fact, by using whole living parasites, it was found that artemisinin treatment did not reduce haemozoin content and as a consequence, earlier statements that artemisinin works through inhibiting haemozoin formation were revised [88]. Later studies with 10-deoxodihydroartemisinin, an effective antimalarial but incapable of inhibiting haemozoin formation, also contradicted this heme-as-target theory [89]. 

A modified version of the heme model is that heme is only an activating agent, but not an important target [90]. However, compounds that are hydrolytically stable and inert towards heme were developed, and these compounds are nonetheless associated with superior antimalarial properties [43]. Moreover, Ro40-4388, a protease inhibitor which blocks the first step in haemoglobin degradation, displayed antagonistic effects against chloroquine, whereas neither antagonistic nor synergistic effects between artemisinin and this drug was observed, suggesting that heme binding was not necessary for the antimalarial activity of artemisinin [41]. Very recently, the antimalarial effect of artemisinins was tested under CO instead of O2. The concept is that CO can bind to heme, forming carboxyhemoglobin (CO-Hb-Fe^2+^) or CO-Hb-Fe^2+^ and may shield its interaction with artemisinins. It was found that artemisinins were more effective when cultured under CO whereas heme-inert artemisinin derivatives less affected, suggesting hemoglobin-Fe^2+^ or heme-Fe^2+^ is not involved in the activation of artemisinins as antimalarial agents [91]. The authors proposed that heme’s action may be related to the degradation of artemisinins instead of potentiating its action. 

In addition to heme, activated artemisinin was also demonstrated to alkylate certain specific malarial proteins. When *P. falciparum*-infected blood cells were treated with radiolabeled arteether, dihydroartemisinin or arteflene, SDS-PAGE isolated six specific non-abundant proteins, whereas no proteins were labeled when erythrocytes alone were treated with these drugs [92]. Further investigation identified one of the principal proteins as the Translationally Controlled Tumor Protein (*Pf*TCTP) [93]. Homology modeling was used to build the 3D structure of this protein. Details of artemisinin docking with heme and subsequently the reaction of activated artemisinin with *Pf*TCTP were given [94]. Nevertheless, there is not much functional evidence and therefore the significance of this interaction in the antimalarial action of artemisinin is still questionable.

### 6.3. Mitochondria as the target

Artemisinin can inhibit yeast growth on nonfermentable (such as glycerol or ethanol as the carbon source) media, but not on fermentable (such as glucose) media. Normally, yeast cells prefer glycolysis on fermentable media (YPD), and they can grow even with dysfunctional mitochondria. In the presence of only nonfermentable carbon sources, functional mitochondria are necessary for their growth. The potent inhibition of yeast on nonfermentable media, but none at all on fermentable media, strongly suggest that artemisinin specifically interferes with yeast mitochondrial functions to suppress its growth [95]. Deletion of *NDE1* or *NDI1*, which encodes external or internal NADH dehydrogenases in the yeast mitochondrial respiration chain reduces artemisinin susceptibility, whereas overexpression resulted in increased sensitivity. Mitochondrial depolarization could also be observed upon artemisinin treatment. These data provided compelling evidence that in yeast, artemisinin works through disruption of normal mitochondrial functions. 

It remains to be seen whether this mitochondrial disruptive event is direct or not. It is conceivable that loss of the mitochondrial function could be a direct event by artemisinin or secondary to another disturbance occurring somewhere else other than mitochondria, but with dysfunctional mitochondria as the readout. For example, mutation in some genes whose products reside in Golgi or vacuoles can affect mitochondrial functions. More crucially, whether or not malaria mitochondria are impacted by artemisinins as yeast mitochondria waits to be seen, *i.e.*, do artemisinins affect malaria parasites similarly as yeast? Finally, because all cell deaths are eventually associated with mitochondrial depolarization, it will also be important to demonstrate that after artemisinin treatment, loss of membrane potential not only happens in malaria, but also is not secondary to cell death.

Functional mitochondrion is indispensable for the growth of *P. falciparum* [96]. Atovaquone, an antimalarial, acts by inhibiting complex III activity [97]. Similarities exist between yeast and *P. falciparum* mitochondrial electron transport chains. For example, BLAST with yeast NDI1 protein in the parasite’s genome led to the finding of one homologous sequence, whereas no such sequence was discovered in all metazoan genomes. In mammals, the counterpart version is a complex (complex I) composed of more than 40 subunits. If iron is indeed involved in the action of artemisinin, in the mitochondrial model the iron’s participation, direct or indirect, could be contemplated as its key involvement in the electron transport chain. In this regard, it would also be interesting to see whether artemisone, a derivative of artemisinin much resistant to ferrous catalysis, acts similarly in yeast. 

According to the mitochondrial model, inactivation of mitochondrial functions by artemisinins is not through inactivating a specific protein target, but instead, may rely on the production of ROS or more general free radicals, a nonspecific damaging agent. Because mammalian cells are not affected by artemisinin, it is speculated that the specificity of action is through specific activation of artemisinin in yeast and malaria whereas mammalian mitochondria may not be able to initiate the action of the drug. But then, one important question is what factors affect the activation of artemisinin? In other words, why are yeast and malarial mitochondria able to properly activate artemisinin while other cells are not? Is it because of the lack of certain factors in these cells or alternatively, spatial hindrance or other causes that prevent the activation of artemisinin?

One recent report posed a contradictory piece of evidence against the mitochondrial mode of action [69]. It is known that upon treating cultured parasites, artemisinin was shown to be distributed to digestive vacuoles [69], neutral lipids [98] and other cytosolic compartments [41,99] such as mitochondria [100]. When the fluorescent dye LysoSensor Blue and Rhodamine 123 were used to monitor the integrity of food vacuoles and mitochondria respectively, damage by artemisinin to the food vacuole was reported to occur at four hours, when mitochondrial change was not observed, suggesting that the digestive vacuole is an important initial site of endoperoxide antimalarial activity. However, in another report, administration of [3H]-dihydroartemisinin to mice inoculated with *P. berghei* was found to cause mitochondrial swelling as early as 30 min [101]. Other studies also reported mitochondrial morphology changes as an early event [100,102,103]. It is not clear how these differences arise. One possibility is that the sensitivities of different monitoring techniques may be different.

## 7. Mutations Affecting Artemisinin Resistance

One major difficulty in analyzing or evaluating the controversies involving the act of artemisinins is the lack of truly artemisinin-resistant strains. Forty years since the discovery of artemisinin, clinically meaningful drug-resistant strains have yet to be found. There have been some recent alerts and reports about the rise of malaria strains that are more tolerant of artemisinins [104,105,106]. These strains nevertheless are still not completely resistant to artemisinin, although reduced clinical efficacy has been observed. In *S. cerevisiae*, no truly resistant clones were isolated from yeast genome-wide mutant screening. Mutation in *NDE1* or *NDI1* confers slightly less than 100-fold more survival under a normally suppressive concentration for normal strains, but these strains can still be effectively inhibited by increasing the drug concentration a few times. Close yeast homologues of proposed candidates such as *TCTP*, *Pfmdr1*, *Pfcrt*, *Pfatp6* genes were also tested for their effects on artemisinin susceptibility [107]. None turned out to be relevant in terms of modulating artemisinin resistance, although it has been reported that variations of *Pfmdr1, Pfcrt and Pfatp6* genes are associated with slightly altered artemisinin susceptibility in malarial field studies [108,109,110]. Consistently, research conducted in Afonso’s group [111] reported that *P. chabaudi chabaudi* with increased resistance to artemisinins contained no changes in the sequence or the copy number of candidate genes such as *Pfatp6*, *TCTP* or *Pfmdr1*. 

Via screening with [112] or without [113] mutagens, *in vitro* resistance of laboratory manipulated malaria parasites to artemisinin were examined. However, in none of these was artemisinin resistance genetically stable and transmissible [111]. The difficulty in developing a true resistance (or qualitative resistance) is compatible with the idea that no single and specific gene is targeted by artemisinin. Although better to be cautious, it may be true that significant resistance to artemisinin may have difficulty naturally arising. Several reasons for the delay of the appearance of resistance have been discussed [114]. Nevertheless, it should be noted that contribution to this property should also be attributed to the chemical and biological characteristics of artemisinins and their chemical analogues. 

## 8. Conclusions

**Figure 2:**
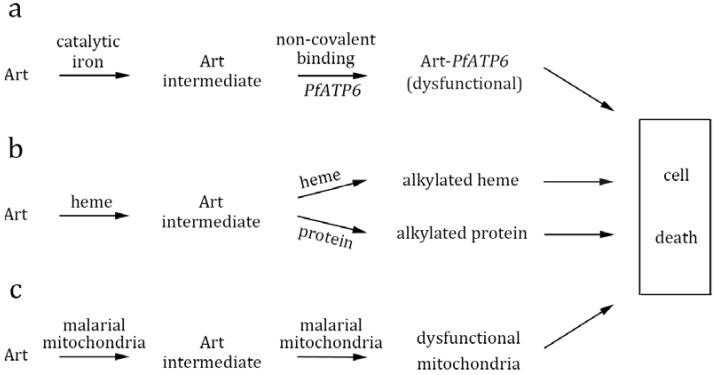
Proposed biological models for the action of artemisinin (Art). (**a**) The *PfATP6* model: artemisinin undergoes activation by reacting with catalytic iron, and after non-covalent specific interaction with *PfATP6*, the formed free radicals then exert irreversible damage to this target protein. (**b**) The heme model: artemisinin is activated by heme, followed by alkylation of heme and/or other malarial proteins. (**c**) The mitochondria model: malarial mitochondria specifically activate artemisinin. The activated artemisinin then induces free radicals production and mitochondrial membrane depolarization. No specific protein targets are implicated.

Three schools of thoughts exist explaining the action of artemisinin (Figure 2). The first one is that artemisinin binds specifically to a target, such as *PfATP6*, in malaria parasites. This mode of action has been supported by some strong evidence, but does not correspond well with chemistry findings on how the backbone and chirality of artemisinins can vary without diminishing their antimalarial activity. The second speculates that artemisinin is uniquely activated by heme in malaria vacuoles. However, some compounds without heme reactivity also displayed potent antimalarial activity. Further, inhibition of heme formation does not prevent the antimalarial action of artemisinin. The third model hypothesizes that artemisinin is activated by malaria mitochondria and then the free radicals non-specifically damage surrounding molecules. This model is demonstrated in yeast, but needs further supporting evidence from malarial studies, as well as some mechanistic insights. Lack of clean genetic evidence certainly poses substantial difficulty to the deciphering of this long standing problem.
